# Supplementary material for: Genome‐wide DNA methylation patterns harbour signatures of hatchling sex and past incubation temperature in a species with environmental sex determination
Source: Mol Ecol. 2022 Sep 5;31(21):5487–505. doi: 10.1111/mec.16670 (PMC9826120; doi:10.1111/mec.16670)
Supplement: Supplementary file 1 — Appendix S1 Table S1 Table S2 Table S3 Table S4 Table S5 Table S6 Table S7 Figure S1 Figure S2 Figure S3 Figure S4 [file MEC-31-5487-s001.pdf]

MOLECULAR ECOLOGY

Supplemental Information for:

Genome-wide DNA methylation patterns harbor signatures of hatchling sex and past incubation temperature in a species with environmental sex determination

Samantha L. Bock, Christopher R. Smaga, Jessica A. McCoy, Benjamin B. Parrott

Table of Contents:

Content	Pages
**Appendix S1.** Supplementary Materials and Methods.	2-3
**Table S1.** Incubation experiments to characterize the American alligator (*Alligator mississippiensis*) temperature-by-sex ratio reaction norm.	4-6
**Table S2.** Sequencing summary for reduced-representation sequencing libraries from hatchling blood cells.	7
**Table S3.** Gene ontology analysis for genes associated with differentially methylated cytosines overlapping promoter regions.	8
**Table S4.** Gene ontology analysis for genes associated with differentially methylated cytosines overlapping promoter regions or gene bodies.	9-13
**Table S5.** Model coefficients for cytosines selected to be included in the predictive model of sex.	14
**Table S6.** Model coefficients for the cytosines selected to be included in the predictive model of incubation temperature.	15-18
**Table S7.** Sequencing summary for reduced-representation sequencing libraries from embryonic gonads.	19
**Figure S1.** Genomic characterization of sex- and temperature-associated differentially methylated cytosines with respect to gene context and CpG density.	20
**Figure S2.** Associations between differentially methylated loci and putative estrogen-response elements.	21
**Figure S3.** Correlations between plasma testosterone concentrations and genome-wide DNA methylation patterns.	22
**Figure S4.** Associations between differentially methylated cytosines and putative androgen response elements.	23

Supplemental Information
Appendix S1. Supplementary Materials and Methods.

Detailed protocol for blood nucleic acid isolation and quantification

DNA was isolated from blood cells using a modified column approach based on the SV total RNA isolation system (Promega). Frozen samples were thawed on ice. To remove RNAlater from the preserved cells, 500 µl of phosphate-buffered saline (PBS) was added to a 200 µl aliquot of each sample and samples were centrifuged at 5000 rcf for 15 minutes at 4˚C (and for an additional 5 min if a cell pellet did not form). Pelleted cells were washed once more with 500 µl of PBS and nucleic acids were isolated according to the protocol reported in (Bae et al., 2021). Specifically, 350 µl of lysis buffer (4 M guanidinium thiocyanate; 0.01 M Tris-HCl, pH 7.5; 2% beta-mercaptoethanol) was added to each sample, and samples were homogenized with a sterile steel bead using a Mini-BeadBeater 24 (BioSpec) at 30 Hz for 2 min. Homogenized samples were then centrifuged for 3 min at 14,000 rcf, and the resulting supernatants transferred to spin columns with silica membranes (Epoch Life Science) and centrifuged for 30 sec at 14,000 rcf to bind DNA to the column. Columns were then washed twice with 700 µl wash buffer (162.8 mM potassium acetate; 27.1 mM Tris-HCl, pH 7.5; diluted with 60% (v/v) ethanol) and DNA was eluted in 60 µl TE buffer (pH 8.0).

Reduced-representation bisulfite sequencing enriches for genomic regions of potential functional significance

A total of 462,236 cytosines was retained in our analyses following filtering, which are referred to as “covered cytosines” or “covered CpGs”. Covered CpGs make up a relatively small proportion (2.2%) of the 21,480,261 CG dinucleotides in the alligator genome. However, as has been previously demonstrated with RRBS techniques, *MspI* digestion followed by size selection resulted in covered CpGs that were enriched in regions of potential functional significance relative to all the CpGs in the genome, including in promoters ($P < 0.0001$; **Figure S1A**) and exons ($P < 0.0001$; **Figure S1A**), as well as CpG islands ($P < 0.0001$), CpG shores ($P < 0.0001$), and CpG shelves ($P < 0.0001$). Covered CpGs were also enriched in intergenic regions ($P < 0.0001$; **Figure S1A**) relative to all CpGs and depleted in introns ($P < 0.0001$; **Figure S1A**) and open sea regions ($P < 0.0001$). Consistent with their distribution with respect to CpG island context, covered CpGs were depleted in genomic tiles of low CpG density (i.e., those containing between 0 and 20 CpGs per 1 kb) and were enriched in genomic tiles of intermediate and high CpG densities (i.e., those containing 20 to 40, 40 to 60, and 60 to 80 CpGs per 1 kb; **Figure S1B**). Covered CpGs were also enriched in genomic regions in proximity to putative EREs ($P < 0.0001$; **Figure S2**) relative to all CpGs in the genome, but not in genomic regions in proximity to putative AREs (**Figure S4**).

Definitions of sex-associated differentially methylated cytosines.

Cytosines that were identified as differentially methylated between all females and males (FvM) *or* between 34.5˚C females and 34.5˚C males ($F^{34.5}vM^{34.5}$) are collectively referred to as “sex-associated DMCs.” If referring to *only* those DMCs identified between all females and males, the term “universal sex-associated DMCs” is used.

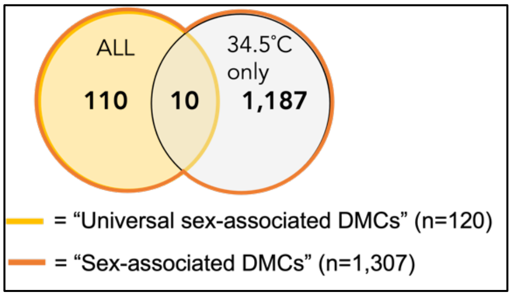

Comparison of predictive models developed with 50:50 split of the data into training and test set

To assess the influence of the size of the training and test sets for the performance of the predictive modeling approach, we split the data into a training set of 12 samples and test set of 12 samples (50:50 split), in addition to the 20-sample training set and 4-sample test set (~80:20 split). For the incubation temperature model, 18 CpGs were selected in the model compared to 24 CpGs in the previous model (~80:20). Of those, eight CpGs were selected to be included in the model across both approaches. The model fit the training set similarly across both approaches with an R-squared over 0.99 and a mean

absolute error of 0.05˚C. For the test set, the R-squared was slightly lower for the 50:50 split with a value of 0.74 compared to the previous 0.81. The mean absolute error, however, was slightly lower for the 50:50 split with a value of 1.00˚C compared to 1.20˚C. For the model to predict sex, 19 CpGs were selected to be included in the model compared to the 20 CpGs in the previous model (~80:20), 16 of which were selected to be included in both models. As with the previous model, sex was correctly identified for all individuals in the training set. For the test set, 11 of 12 (91.7%) individuals were correctly assigned sex compared to 4 of 4 for the previous model. Overall, models based on an ~80:20 split of the data and a 50:50 split of the data tend to perform similarly.

Table S1. Incubation experiments to characterize the American alligator (*Alligator mississippiensis*) temperature-by-sex ratio reaction norm.

Study ID	Reference	Incubation Temperature	# female	# male	% female	Method of sexing	Egg collection and other notes
A	(Joanen, McNease, & Ferguson, 1987)	32.8	0	106	0		
A		31.7	13	38	0.25490196		
A		30.6	19	13	0.59375		
A		29.4	80	0	1		
B	(Janes et al., 2013)	28	10	0	1	Macroscopic inspection (Moore et al., 2008)	Field collection, "late-stage embryos"
B		33	0	10	0		
C	(Lance & Bogart, 1991)	30	25	0	1	Histology	Eggs collected from wild nests within 1-2 days of oviposition; subset injection of vehicle (corn oil)
C		33	0	27	0		
D	(Lance & Bogart, 1992)	30	58	0	1	Histology	Subset injected with vegetable oil
D		33	0	56	0		
E	(Lang & Andrews, 1994)	29	113	0	1	Macroscopic inspection of gonads or genitalia at various ages	Eggs collected from wild nests within 1 week of oviposition
E		30	27	0	1		
E		30.5	27	0	1		
E		31	197	0	1		
E		31.5	125	0	1		
E		32	114	228	0.33333333		
E		32.5	0	122	0		
E		33	0	97	0		
E		33.5	3	16	0.15789474		
E		34	35	19	0.64814815		
E		34.5	14	1	0.93333333		
F	(Milnes et al., 2004)	32	11.62	2.38	0.83	Histology	Eggs collected from wild nests within 2 weeks of oviposition; unsure of exact sample sizes, values reported are the max possible based on methods
F		33.5	0	14	0		
G	(Milnes et al., 2005)	32	5	5	0.5	Histology	Eggs collected from wild nests within 2 weeks of oviposition
G		33.5	0	10	0		
H	(Moore et al., 2010)	30	12	0	1	Macroscopic inspection of gonad, presence of oviduct at 13 months	Included eggs collected from Lake Woodruff and Lake Apopka; sex ratio also includes the effect of survival to 13 months
H		33.5	0	13	0		
H		32	8	19	0.2962963		
I	(Guillette Jr. et al., 1994)	30.5	24	14	0.63157895	Histology	Average age of egg at collection was 12 days; included individuals from both Lake Apopka and Lake Woodruff
J	(Conley et al., 1997)	31	14	0	1	Histology (between stage 25 and hatching)	Data included here are from 1995 study
J		33	0	10	0		
J		34	11	12	0.47826087		
J		34.2	5	0	1		
K	(Ferguson & Joanen, 1983)	30	97	0	1	Histology at day 60 of incubation	Eggs collected within 10hr of oviposition
K		32	85	13	0.86734694		
K		34	0	94	0		
L	(Deeming & Ferguson, 1991)	30	19	0	1	Macroscopic inspection of gonad at day 60 of incubation	Only included control individuals from each study year
L		33	0	19	0		
M	(Bock et al., 2021)	30	68	1	0.98550725		Included all treatment groups
M		33.5	1	62	0.01587302		

Study ID	Reference	Incubation Temperature	# female	# male	% female	Method of sexing	Egg collection and other notes
M		31.2	95	0	1	Macroscopic inspection of gonad, presence of oviduct at 10 days post-hatch	
N	(McCoy et al., 2016)	30	19	0	1	AMH and CYP19A1 expression	All eggs collected from wild nests at Lake Apopka, FL
N		32	41	15	0.73214286		
N		33.5	4	16	0.2		
N		34.5	12	46	0.20689655		

References:

(A) Joanen, T., McNease, L., & Ferguson, M. W. J. (1987). The effects of egg incubation temperature on post-hatching growth of American alligators. In *Wildlife Management: Crocodiles and Alligators* (ed. Webb, G. J. W., Manolis, S.C., & Whitehead, P. J.), pp. 533-537. New South Wales, Australia: Surrey Beatty and Sons.

(B) Janes, D. E., Elsey, R. M., Langan, E. M., Valenzuela, N., & Edwards, S.V. (2013). Sex-biased expression of sex-differentiating genes FOXL2 and FGF9 in American alligators, *Alligator mississippiensis*. *Sexual Development*, *7*, 253–260.

(C) Lance, V. A., & Bogart, M. H. (1991). Tamoxifen 'sex reverses' alligator embryos at male producing temperature, but is an antiestrogen in female hatchlings. *Experientia*, *47*, 263–266.

(D) Lance, V. A., & Bogart, M. H. (1992). Disruption of ovarian development in alligator embryos treated with an aromatase inhibitor. *General and Comparative Endocrinology*, *86*, 59–71.

(E) Lang, J. W., & Andrews, H. V. (1994). Temperature-dependent sex determination in crocodilians. *Journal of Experimental Zoology*, *270*(1), 28–44.

(F) Milnes, M. R., Allen, D., Bryan, T. A., Sedacca, C. D., & Guillette, L. J. (2004). Developmental effects of embryonic exposure to toxaphene in the American alligator (*Alligator mississippiensis*). *Comparative Biochemistry and Physiology Part C: Toxicology & Pharmacology*, *138*, 81–87.

(G) Milnes, M. R., Bryan, T. A., Medina, J. G., Gunderson, M. P., & Guillette, L. J. Jr (2005). Developmental alterations as a result of in ovo exposure to the pesticide metabolite p,p′-DDE in *Alligator mississippiensis*. *General and Comparative Endocrinology*, *144*, 257–263.

(H) Moore, B. C., Milnes, M. R., Kohno, S., Katsu, Y., Iguchi, T., & Guillette, L. J. (2010). Influences of sex, incubation temperature, and environmental quality on gonadal estrogen and androgen receptor messenger RNA expression in juvenile American alligators (*Alligator mississippiensis*). *Biology of Reproduction*, *82*, 194–201.

(I) Guillette, L. J. Jr, Gross, T. S., Masson, G. R., Matter, J. M., Percival, H. F., & Woodward, A. R. (1994). Developmental abnormalities of the gonad and abnormal sex hormone concentrations in juvenile alligators from contaminated and control lakes in Florida. *Environmental Health Perspectives*, *102*, 680–688.

(J) Conley, A. J., Elf, P., Corbin, C. J., Dubowsky, S., Fivizzani, A., & Lang, J. W. (1997). Yolk steroids decline during sexual differentiation in the alligator. *General and Comparative Endocrinology*, *107*, 191–200.

(K) Ferguson, M. W., & Joanen, T. (1983). Temperature-dependent sex determination in *Alligator mississippiensis*. *Journal of Zoology*, *200*, 143–177.

(L) Deeming, D. C., & Ferguson, M. W. J. (1991). Reduction in eggshell conductance to respiratory gases has no effect on sex determination in *Alligator mississippiensis*. *Copeia*, *1991*, 240–243.

(M) Bock, S. L., Hale, M. D., Rainwater, T. R., Wilkinson, P. M., & Parrott, B. B. 2021. Incubation temperature and maternal resource provisioning, but not contaminant exposure, shape hatchling phenotypes in a species with temperature-dependent sex determination. *Biological Bulletin*, *241*(1), 43-54.

(N) McCoy, J. A., Hamlin, H. J., Thayer, L. A., Guillette, L. J., & Parrott, B. B. (2016). The influence of thermal signals during embryonic development on intrasexual and sexually dimorphic gene expression and circulating steroid hormones in American alligator hatchlings (*Alligator mississippiensis*). *General and Comparative Endocrinology*, *238*, 47–54.

Table S2. Sequencing summary for reduced-representation sequencing libraries from hatchling blood cells.

Sample	Temperature	Sex	Clutch	Total reads	Total aligned reads	Percent alignment	Bisulfite conversion efficiency
1	30	F	AP-03	6525084	4213047	64.5669389	99.6197
2	33.5	M	AP-06	10336198	6617520	64.0227674	99.3485
3	33.5	M	AP-08	7501614	4905246	65.3892082	99.1967
4	34.5	F	AP-06	10819612	6718596	62.0964597	99.5743
5	33.5	M	AP-04	8676405	5430747	62.5921335	99.5053
6	34.5	M	AP-06	8170904	5098412	62.3971595	99.7712
7	30	F	AP-04	9064806	5734847	63.2649722	99.6243
8	34.5	M	AP-03	9038702	5882088	65.0766891	99.3175
9	30	F	AP-03	10732905	6762810	63.0100611	99.8536
10	30	F	AP-06	9106305	5885739	64.6336687	99.896
11	33.5	M	AP-04	7543166	4976851	65.9782776	99.3638
12	34.5	M	AP-03	10512637	6545212	62.2604205	99.5369
13	33.5	M	AP-03	7589151	4805508	63.3207588	99.6802
14	33.5	M	AP-03	8129634	5234303	64.3854693	99.4774
15	30	F	AP-08	8422219	5270330	62.5765015	99.4653
16	33.5	M	AP-08	7724544	4807692	62.2391691	99.9292
17	34.5	F	AP-03	8734654	5610949	64.2377935	99.8738
18	33.5	M	AP-05	8310169	5387366	64.8285973	99.1258
19	30	F	AP-05	9557288	6006117	62.8433192	99.889
20	30	F	AP-08	8069098	5006391	62.043998	99.8269
21	34.5	F	AP-04	7613576	4990415	65.5462689	99.3034
22	34.5	F	AP-03	8080586	4994461	61.8081535	99.2036
23	30	F	AP-04	7639275	4849357	63.479283	99.5237
24	34.5	M	AP-04	8965500	5765134	64.3035414	99.7648

Table S3. Gene ontology analysis for genes associated with differentially methylated cytosines overlapping promoter regions.

**Sex-associated DMCs** (across all or within 34.5˚C individuals)			
Source	GO term name	GO term ID	Benjamini-Hochberg FDR
GO:MF	DNA binding domain binding	GO:0050692	0.099478029
**Universal sex-associated DMCs** (across all individuals)			
Source	GO term name	GO term ID	Benjamini-Hochberg FDR
NA	NA	NA	NA
**Temperature-associated DMCs**			
Source	GO term name	GO term ID	Benjamini-Hochberg FDR
GO:CC	integral component of synaptic membrane	GO:0099699	0.066156435
GO:CC	cell-cell contact zone	GO:0044291	0.066156435
GO:CC	early phagosome	GO:0032009	0.066156435
GO:CC	intrinsic component of synaptic membrane	GO:0099240	0.067585178

Table S4. Gene ontology analysis for genes associated with differentially methylated cytosines overlapping promoter regions or gene bodies.

**Sex-associated DMCs** (across all or within 34.5˚C individuals)			
Source	GO term name	GO term ID	Benjamini-Hochberg FDR
GO:MF	phosphatidylinositol phospholipase C activity	GO:0004435	0.046729221
GO:MF	phospholipase C activity	GO:0004629	0.046729221
GO:MF	ionotropic glutamate receptor activity	GO:0004970	0.091253906
GO:MF	glutamate receptor activity	GO:0008066	0.091253906
GO:MF	signaling receptor activity	GO:0038023	0.091253906
GO:MF	molecular transducer activity	GO:0060089	0.091253906
GO:BP	system development	GO:0048731	4.67E-05
GO:BP	multicellular organism development	GO:0007275	9.97E-05
GO:BP	anatomical structure development	GO:0048856	9.97E-05
GO:BP	developmental process	GO:0032502	9.97E-05
GO:BP	nervous system development	GO:0007399	9.97E-05
GO:BP	animal organ development	GO:0048513	0.000461474
GO:BP	cellular developmental process	GO:0048869	0.00103919
GO:BP	modulation of excitatory postsynaptic potential	GO:0098815	0.001098492
GO:BP	cell differentiation	GO:0030154	0.001444742
GO:BP	multicellular organismal process	GO:0032501	0.001444742
GO:BP	cell development	GO:0048468	0.002087083
GO:BP	glutamate receptor signaling pathway	GO:0007215	0.002290445
GO:BP	generation of neurons	GO:0048699	0.002290445
GO:BP	neurogenesis	GO:0022008	0.002356907
GO:BP	cell junction organization	GO:0034330	0.003451143
GO:BP	synapse assembly	GO:0007416	0.003451143
GO:BP	establishment of cell polarity	GO:0030010	0.003600573
GO:BP	synapse organization	GO:0050808	0.004147013
GO:BP	anatomical structure morphogenesis	GO:0009653	0.004455126
GO:BP	cell adhesion	GO:0007155	0.006689459
GO:BP	biological adhesion	GO:0022610	0.006689459
GO:BP	neuron differentiation	GO:0030182	0.006689459
GO:BP	cell junction assembly	GO:0034329	0.006689459
GO:BP	movement of cell or subcellular component	GO:0006928	0.006689459
GO:BP	regulation of postsynaptic membrane potential	GO:0060078	0.00887211
GO:BP	positive regulation of nervous system development	GO:0051962	0.009089329
GO:BP	regulation of endothelial cell migration	GO:0010594	0.009089329
GO:BP	endothelial cell migration	GO:0043542	0.011361955
GO:BP	modulation of chemical synaptic transmission	GO:0050804	0.019360829
GO:BP	plasma membrane bounded cell projection morphogenesis	GO:0120039	0.019360829
GO:BP	regulation of trans-synaptic signaling	GO:0099177	0.019360829
GO:BP	neuron projection morphogenesis	GO:0048812	0.019360829
GO:BP	circulatory system development	GO:0072359	0.019360829
GO:BP	cell projection morphogenesis	GO:0048858	0.019360829

Source	GO term name	GO term ID	Benjamini-Hochberg FDR
GO:BP	regulation of membrane potential	GO:0042391	0.019360829
GO:BP	system process	GO:0003008	0.019554585
GO:BP	neuron development	GO:0048666	0.021614364
GO:BP	locomotion	GO:0040011	0.021697904
GO:BP	ionotropic glutamate receptor signaling pathway	GO:0035235	0.02197245
GO:BP	axon development	GO:0061564	0.02197245
GO:BP	ligand-gated ion channel signaling pathway	GO:1990806	0.02197245
GO:BP	positive regulation of excitatory postsynaptic potential	GO:2000463	0.02197245
GO:BP	cell part morphogenesis	GO:0032990	0.023978992
GO:BP	nervous system process	GO:0050877	0.023978992
GO:BP	blood vessel morphogenesis	GO:0048514	0.023978992
GO:BP	regulation of nervous system development	GO:0051960	0.023978992
GO:BP	regulation of cell junction assembly	GO:1901888	0.025475418
GO:BP	neuron recognition	GO:0008038	0.027444083
GO:BP	regulation of plasma membrane bounded cell projection organization	GO:0120035	0.027444083
GO:BP	regulation of nervous system process	GO:0031644	0.027444083
GO:BP	protein sulfation	GO:0006477	0.027444083
GO:BP	regulation of synapse organization	GO:0050807	0.030946139
GO:BP	cellular component morphogenesis	GO:0032989	0.035379731
GO:BP	cell morphogenesis involved in neuron differentiation	GO:0048667	0.035379731
GO:BP	axonogenesis	GO:0007409	0.037290698
GO:BP	neuron projection organization	GO:0106027	0.037290698
GO:BP	regulation of synapse assembly	GO:0051963	0.037665841
GO:BP	branching involved in mammary gland duct morphogenesis	GO:0060444	0.03869607
GO:BP	regulation of cell projection organization	GO:0031344	0.040092606
GO:BP	regulation of synapse structure or activity	GO:0050803	0.040931059
GO:BP	cell projection organization	GO:0030030	0.048480681
GO:BP	cell recognition	GO:0008037	0.052433926
GO:BP	excitatory postsynaptic potential	GO:0060079	0.052479576
GO:BP	establishment or maintenance of cell polarity	GO:0007163	0.056766179
GO:BP	regulation of neuron projection development	GO:0010975	0.056766179
GO:BP	neuron projection development	GO:0031175	0.056766179
GO:BP	plasma membrane bounded cell projection organization	GO:0120036	0.057437684
GO:BP	positive regulation of neurogenesis	GO:0050769	0.057437684
GO:BP	regulation of regulated secretory pathway	GO:1903305	0.063430303
GO:BP	chemical synaptic transmission, postsynaptic	GO:0099565	0.064017036
GO:BP	neuron projection guidance	GO:0097485	0.064017036
GO:BP	axon guidance	GO:0007411	0.064017036
GO:BP	tube morphogenesis	GO:0035239	0.064017036
GO:BP	neuromuscular process controlling balance	GO:0050885	0.067510989
GO:BP	vocalization behavior	GO:0071625	0.06884488
GO:BP	cell motility	GO:0048870	0.06884488

Source	GO term name	GO term ID	Benjamini-Hochberg FDR
GO:BP	localization of cell	GO:0051674	0.06884488
GO:BP	regulation of exocytosis	GO:0017157	0.06884488
GO:BP	cell surface receptor signaling pathway	GO:0007166	0.069936321
GO:BP	blood vessel development	GO:0001568	0.072488886
GO:BP	tube development	GO:0035295	0.072488886
GO:BP	vasculature development	GO:0001944	0.072707903
GO:BP	central nervous system development	GO:0007417	0.072707903
GO:BP	cell migration	GO:0016477	0.073692838
GO:BP	mammary gland branching involved in pregnancy	GO:0060745	0.073942135
GO:BP	negative regulation of toll-like receptor signaling pathway	GO:0034122	0.075190437
GO:BP	regulation of system process	GO:0044057	0.076765406
GO:BP	postsynapse organization	GO:0099173	0.076765406
GO:BP	positive regulation of endothelial cell migration	GO:0010595	0.076765406
GO:BP	positive regulation of blood vessel endothelial cell migration	GO:0043536	0.076765406
GO:BP	regulation of epithelial cell migration	GO:0010632	0.085419205
GO:BP	brain development	GO:0007420	0.085419205
GO:BP	action potential	GO:0001508	0.088182096
GO:BP	neuromuscular process	GO:0050905	0.090126529
GO:BP	tissue migration	GO:0090130	0.092197216
GO:BP	syncytium formation by plasma membrane fusion	GO:0000768	0.09630765
GO:BP	positive regulation of cell projection organization	GO:0031346	0.09630765
GO:BP	cell-cell fusion	GO:0140253	0.09630765
GO:BP	chemotaxis	GO:0006935	0.096934946
GO:BP	cell morphogenesis involved in differentiation	GO:0000904	0.096934946
GO:BP	regulation of cellular component biogenesis	GO:0044087	0.096934946
GO:BP	neuron cell-cell adhesion	GO:0007158	0.09719147
GO:BP	postsynapse assembly	GO:0099068	0.09719147
GO:CC	anchored component of membrane	GO:0031225	5.28E-05
GO:CC	cell periphery	GO:0071944	0.000783107
GO:CC	neuron projection	GO:0043005	0.001236169
GO:CC	cell projection	GO:0042995	0.001236169
GO:CC	plasma membrane	GO:0005886	0.001236169
GO:CC	neuronal cell body	GO:0043025	0.001240491
GO:CC	plasma membrane bounded cell projection	GO:0120025	0.001564383
GO:CC	receptor complex	GO:0043235	0.001807446
GO:CC	cell junction	GO:0030054	0.002505658
GO:CC	cell body	GO:0044297	0.002505658
GO:CC	somatodendritic compartment	GO:0036477	0.003609509
GO:CC	dendrite	GO:0030425	0.005212251
GO:CC	dendritic tree	GO:0097447	0.005251196
GO:CC	cell-cell junction	GO:0005911	0.008041091
GO:CC	axon	GO:0030424	0.017198764
GO:CC	synapse	GO:0045202	0.019393832
GO:CC	intrinsic component of membrane	GO:0031224	0.021794879

Source	GO term name	GO term ID	Benjamini-Hochberg FDR
GO:CC	external encapsulating structure	GO:0030312	0.022018343
GO:CC	extracellular matrix	GO:0031012	0.022018343
GO:CC	intrinsic component of plasma membrane	GO:0031226	0.022018343
GO:CC	postsynaptic membrane	GO:0045211	0.023984963
GO:CC	neuron to neuron synapse	GO:0098984	0.025952643
GO:CC	asymmetric synapse	GO:0032279	0.025952643
GO:CC	integral component of plasma membrane	GO:0005887	0.035700497
GO:CC	cell surface	GO:0009986	0.035700497
GO:CC	postsynaptic density	GO:0014069	0.035700497
GO:CC	muscle cell projection	GO:0036194	0.035700497
GO:CC	muscle cell projection membrane	GO:0036195	0.035700497
GO:CC	CNTFR-CLCF1 complex	GO:0097059	0.035700497
GO:CC	synaptic membrane	GO:0097060	0.035700497
GO:CC	anchoring junction	GO:0070161	0.040184607
GO:CC	cytoskeleton	GO:0005856	0.045271109
GO:CC	potassium channel complex	GO:0034705	0.047773629
GO:CC	sodium channel complex	GO:0034706	0.047773629
GO:CC	cation channel complex	GO:0034703	0.060547769
GO:CC	postsynaptic specialization	GO:0099572	0.063844236
GO:CC	plasma membrane region	GO:0098590	0.063844236
GO:CC	postsynaptic specialization membrane	GO:0099634	0.063844236
GO:CC	kainate selective glutamate receptor complex	GO:0032983	0.078195412
GO:CC	cytolytic granule	GO:0044194	0.078195412
GO:CC	cytoplasmic region	GO:0099568	0.079007217
GO:CC	postsynapse	GO:0098794	0.080518304
GO:CC	plasma membrane protein complex	GO:0098797	0.088151107
GO:CC	microfibril	GO:0001527	0.094177952
**Universal sex-associated DMCs** (across all individuals)			
Source	GO term name	GO term ID	Benjamini-Hochberg FDR
GO:CC	CNTFR-CLCF1 complex	GO:0097059	0.005657706
**Temperature-associated DMCs**			
Source	GO term name	GO term ID	Benjamini-Hochberg FDR
GO:BP	system development	GO:0048731	0.034970995
GO:BP	signaling	GO:0023052	0.090335271
GO:BP	movement of cell or subcellular component	GO:0006928	0.093572399
GO:BP	regulation of signaling	GO:0023051	0.098392685
GO:BP	cell migration	GO:0016477	0.098392685
GO:BP	regulation of cell communication	GO:0010646	0.098392685
GO:BP	multicellular organism development	GO:0007275	0.098392685
GO:BP	signal transduction	GO:0007165	0.098392685
GO:BP	cell communication	GO:0007154	0.098392685
GO:BP	vasculature development	GO:0001944	0.098392685
GO:BP	blood vessel development	GO:0001568	0.098392685
GO:BP	cellular response to stimulus	GO:0051716	0.098392685

Source	GO term name	GO term ID	Benjamini-Hochberg FDR
GO:CC	cell junction	GO:0030054	0.008213443
GO:CC	cell periphery	GO:0071944	0.008213443
GO:CC	plasma membrane	GO:0005886	0.032772209

Table S5. Model coefficients for cytosines selected to be included in the predictive model of sex.

seqnames	starts	ends	coefficients
NW_017707593.1	91913	91913	-0.0356773
NW_017707836.1	1654499	1654499	0.03212573
NW_017707977.1	543656	543656	0
NW_017708230.1	3394	3394	-0.0089074
NW_017708561.1	39079	39079	0.01909979
NW_017708944.1	151	151	-0.011266
NW_017709083.1	3729449	3729449	-0.0122084
NW_017709336.1	14554887	14554887	-0.0086496
NW_017709336.1	2808872	2808872	0.04981291
NW_017709809.1	4507591	4507591	-0.0263089
NW_017709848.1	131158	131158	0
NW_017710078.1	1083668	1083668	-0.0258123
NW_017710078.1	1172445	1172445	0
NW_017710621.1	24066900	24066900	0.02225572
NW_017710865.1	13502	13502	0.03607998
NW_017710965.1	10634	10634	0.06808733
NW_017712138.1	20423104	20423104	-0.0342119
NW_017712461.1	405501	405501	0.01471531
NW_017713645.1	39474677	39474677	-0.0278214
NW_017713709.1	15783590	15783590	-0.023263
NW_017713972.1	4410508	4410508	0.0220656
NW_017714183.1	13535814	13535814	-0.0080856
NW_017714267.1	48405077	48405077	-0.0087841
**intercept**			-3.3731516

Table S6. Model coefficients for the cytosines selected to be included in the predictive model of incubation temperature.

seqnames	starts	ends	coefficients
NW_017707537.1	1156821	1156821	0
NW_017707575.1	664576	664576	0
NW_017707593.1	31566915	31566915	0
NW_017707680.1	97612	97612	0
NW_017707689.1	612408	612408	0
NW_017707789.1	246112	246112	0
NW_017707836.1	13872742	13872742	0
NW_017707888.1	59383	59383	0
NW_017707888.1	6175388	6175388	0
NW_017707901.1	19373924	19373924	0
NW_017707901.1	20157398	20157398	0
NW_017707947.1	55173633	55173633	0
NW_017707977.1	2267140	2267140	-0.0124876
NW_017708049.1	7444667	7444667	0
NW_017708054.1	4751706	4751706	0
NW_017708115.1	890584	890584	0
NW_017708254.1	362681	362681	-0.0163736
NW_017708254.1	522064	522064	0
NW_017708373.1	2095	2095	0
NW_017708483.1	12588	12588	0
NW_017708551.1	15179	15179	0
NW_017708624.1	12784596	12784596	0
NW_017708624.1	15345883	15345883	0
NW_017708672.1	2204425	2204425	0
NW_017708718.1	50920	50920	-0.0036008
NW_017708734.1	52212	52212	0
NW_017708765.1	4254832	4254832	0
NW_017708765.1	5603885	5603885	0
NW_017708776.1	109935	109935	0
NW_017708796.1	556167	556167	0
NW_017708899.1	10669901	10669901	0
NW_017708899.1	14520000	14520000	0
NW_017708899.1	15794844	15794844	0
NW_017709017.1	1002275	1002275	0
NW_017709017.1	729544	729544	0
NW_017709029.1	144476	144476	0
NW_017709042.1	22962869	22962869	0
NW_017709065.1	706150	706150	0
NW_017709065.1	800711	800711	0
NW_017709067.1	31571	31571	0
NW_017709154.1	15587	15587	0
NW_017709210.1	101550	101550	0
NW_017709210.1	151409	151409	0
NW_017709336.1	13952461	13952461	0

seqnames	starts	ends	coefficients
NW_017709344.1	528603	528603	0
NW_017709480.1	8165125	8165125	0
NW_017709599.1	10555075	10555075	0
NW_017709599.1	11694301	11694301	0
NW_017709599.1	11803781	11803781	0
NW_017709599.1	7424511	7424511	-0.0014596
NW_017709599.1	8690707	8690707	0
NW_017709854.1	27383056	27383056	0
NW_017709984.1	695115	695115	0.00080832
NW_017710057.1	260299	260299	0
NW_017710078.1	1172445	1172445	0
NW_017710164.1	90031	90031	0
NW_017710321.1	10692071	10692071	0
NW_017710432.1	3336829	3336829	0
NW_017710448.1	397324	397324	0
NW_017710448.1	821723	821723	0
NW_017710466.1	1741	1741	-5.62E-06
NW_017710504.1	52087	52087	0
NW_017710534.1	5333531	5333531	-0.003189
NW_017710560.1	331268	331268	-0.0006225
NW_017710621.1	23656719	23656719	0
NW_017710746.1	4471645	4471645	0
NW_017710946.1	3320418	3320418	0
NW_017710965.1	10634	10634	0.02114739
NW_017710976.1	756238	756238	0.04154243
NW_017711041.1	3475323	3475323	0
NW_017711054.1	18358	18358	0
NW_017711096.1	25640420	25640420	6.32E-05
NW_017711096.1	28717706	28717706	0
NW_017711096.1	5619169	5619169	0
NW_017711201.1	646956	646956	0
NW_017711293.1	1811343	1811343	0
NW_017711293.1	464681	464681	0
NW_017711319.1	5863921	5863921	0
NW_017711344.1	7021	7021	0
NW_017711346.1	244563	244563	0
NW_017711487.1	10068264	10068264	-0.0051853
NW_017711487.1	2051686	2051686	0
NW_017711487.1	4940281	4940281	0
NW_017711487.1	5148758	5148758	0
NW_017711527.1	11969347	11969347	0
NW_017711567.1	1335170	1335170	0
NW_017711568.1	10926618	10926618	0
NW_017711568.1	18109023	18109023	-0.0020448
NW_017711692.1	15736498	15736498	0
NW_017711861.1	5666	5666	0
NW_017711921.1	1469	1469	0

seqnames	starts	ends	coefficients
NW_017711921.1	1474	1474	0
NW_017711985.1	207462	207462	0
NW_017712118.1	16197695	16197695	0
NW_017712138.1	116740	116740	0
NW_017712138.1	20423104	20423104	0
NW_017712138.1	8576097	8576097	0.01340404
NW_017712138.1	959869	959869	0
NW_017712186.1	6018016	6018016	-0.0139219
NW_017712194.1	160546	160546	0
NW_017712303.1	2366038	2366038	0
NW_017712464.1	9557375	9557375	0
NW_017712924.1	3348061	3348061	0
NW_017713026.1	1944	1944	0
NW_017713083.1	108585	108585	0
NW_017713083.1	1856586	1856586	0
NW_017713165.1	343	343	0
NW_017713334.1	62391	62391	0
NW_017713446.1	6101896	6101896	0
NW_017713475.1	1947470	1947470	0
NW_017713485.1	320211	320211	0
NW_017713513.1	11479708	11479708	0.01415717
NW_017713513.1	2852287	2852287	0
NW_017713516.1	444921	444921	0
NW_017713629.1	17031206	17031206	0.01315481
NW_017713629.1	18243123	18243123	0
NW_017713629.1	18712057	18712057	0.00178697
NW_017713629.1	19278690	19278690	0
NW_017713629.1	19583010	19583010	0.01283963
NW_017713645.1	17492529	17492529	0
NW_017713645.1	9641978	9641978	0
NW_017713697.1	15320021	15320021	0.0047776
NW_017713697.1	20082957	20082957	0
NW_017713697.1	29698341	29698341	0
NW_017713697.1	4576647	4576647	0
NW_017713700.1	1069506	1069506	0.00399025
NW_017713703.1	317477	317477	0
NW_017713709.1	1234746	1234746	0
NW_017713709.1	2137652	2137652	0
NW_017713709.1	21601852	21601852	0
NW_017713709.1	9212745	9212745	0
NW_017713772.1	18699692	18699692	0
NW_017713772.1	6328963	6328963	0.00382647
NW_017713851.1	22425584	22425584	0
NW_017713894.1	2814789	2814789	0
NW_017713955.1	3014	3014	0
NW_017713998.1	7077131	7077131	0
NW_017714045.1	91353	91353	0

**seqnames**	**starts**	**ends**	**coefficients**
NW_017714045.1	9230558	9230558	0.00635438
NW_017714054.1	463859	463859	0
NW_017714106.1	1217490	1217490	0
NW_017714180.1	1648	1648	0
NW_017714194.1	16670684	16670684	0
NW_017714219.1	89786	89786	0
NW_017714267.1	42786365	42786365	0
NW_017714267.1	49033156	49033156	0
NW_017714270.1	352270	352270	0
NW_017714436.1	1312872	1312872	0
NW_017714440.1	542	542	0
NW_017714442.1	193621	193621	0.0122612
NW_017714443.1	762448	762448	0
NW_017714467.1	842	842	0
**intercept**			2.40E+01

Table S7. Sequencing summary for reduced-representation sequencing libraries from embryonic gonads.

Sample	Temperature	Clutch	Total reads	Total aligned reads	Percent alignment	Bisulfite conversion efficiency
1	33.5	WO-03	5929216	3395703	57.2706914	99.9297
3	34.5	WO-10	11151190	6374060	57.1603569	99.5941
4	32	WO-01	14163866	7761664	54.7990499	99.5042
5	29	WO-14	14515134	7901948	54.4393734	99.1275
6	29	WO-03	8532667	4656977	54.578211	99.9642
7	33.5	WO-01	11466703	6132469	53.4806648	99.6724
8	33.5	WO-14	10666274	5542201	51.9600472	99.3716
9	29	WO-14	8563375	4797032	56.0180069	99.9326
10	33.5	WO-14	5772967	3079819	53.3489798	99.2703
11	32	WO-14	8346295	4493569	53.8390867	99.5219
12	33.5	WO-10	8423048	4524574	53.7165881	99.4297
13	34.5	WO-14	6072181	3444702	56.7292378	99.9121
14	34.5	WO-08	6577901	3781251	57.4841579	98.6111
15	32	WO-14	7360671	3942372	53.559954	99.8255
16	33.5	WO-08	6166782	3310883	53.6889905	99.8789
17	29	WO-08	8729572	4972378	56.960158	99.8904
18	29	WO-10	7859923	4401316	55.9969353	99.2152
19	34.5	WO-01	8430493	4806315	57.0110787	99.7643
21	32	WO-08	8992885	4957061	55.1220326	99.5529
22	32	WO-03	8967757	4990198	55.6459993	99.8441
23	29	WO-01	7878311	4475609	56.809245	99.8505
24	32	WO-10	8438401	4815033	57.0609645	99.6923

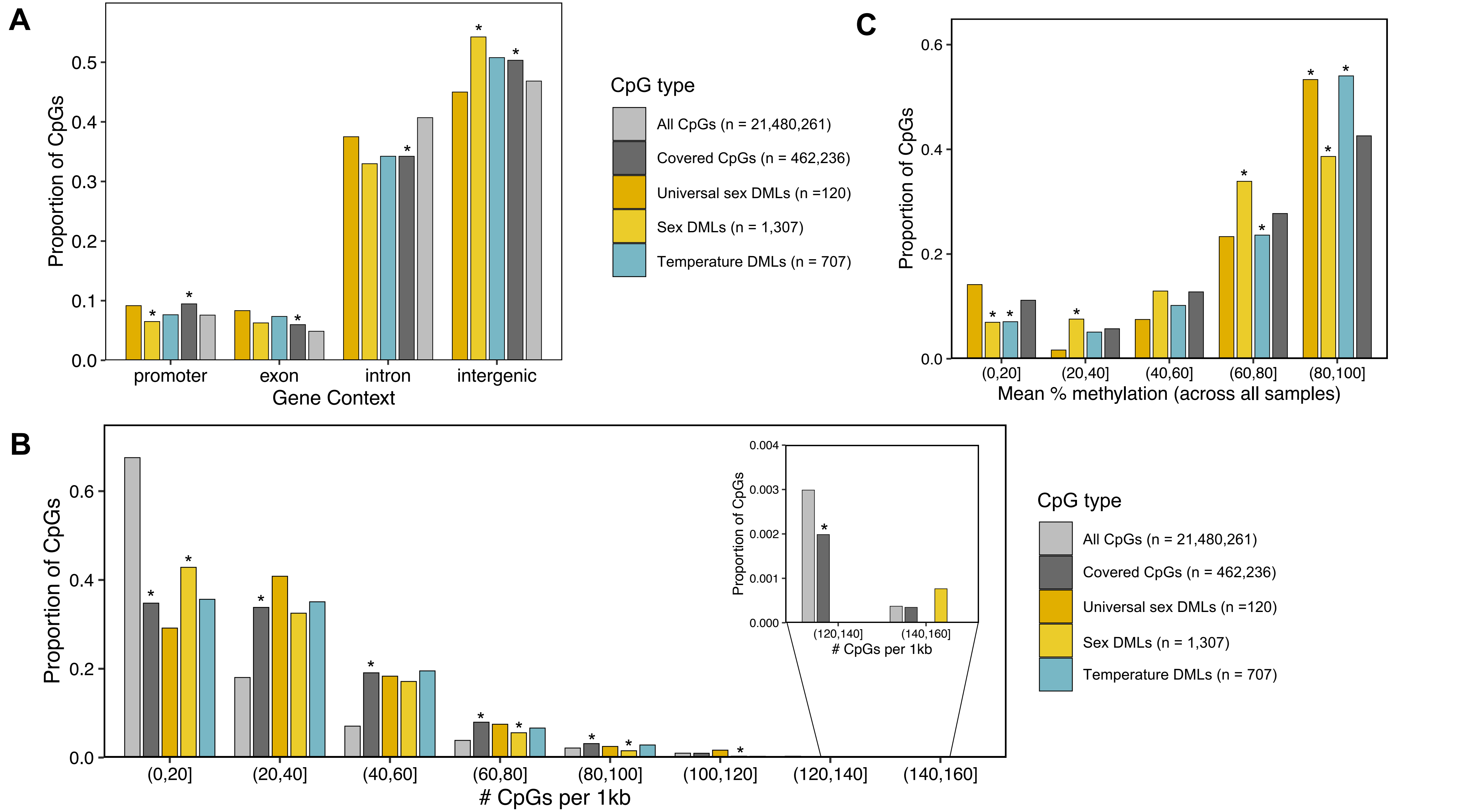

Figure S1. Genomic characterization of sex- and temperature-associated differentially methylated loci. (A) Proportion of CpGs overlapping promoters, exons, introns, and intergenic regions. (B) Proportion of CpGs overlapping 1kb tiles of varying CpG density. (C) Proportion of CpGs with varying mean percent methylation across all samples. Asterisks indicate significant difference in the proportion of DMCs in a context category compared to the covered CpGs or significant difference in the proportion of covered CpGs in a context category compared to all the CpGs in the alligator genome at an FDR threshold of 10%.

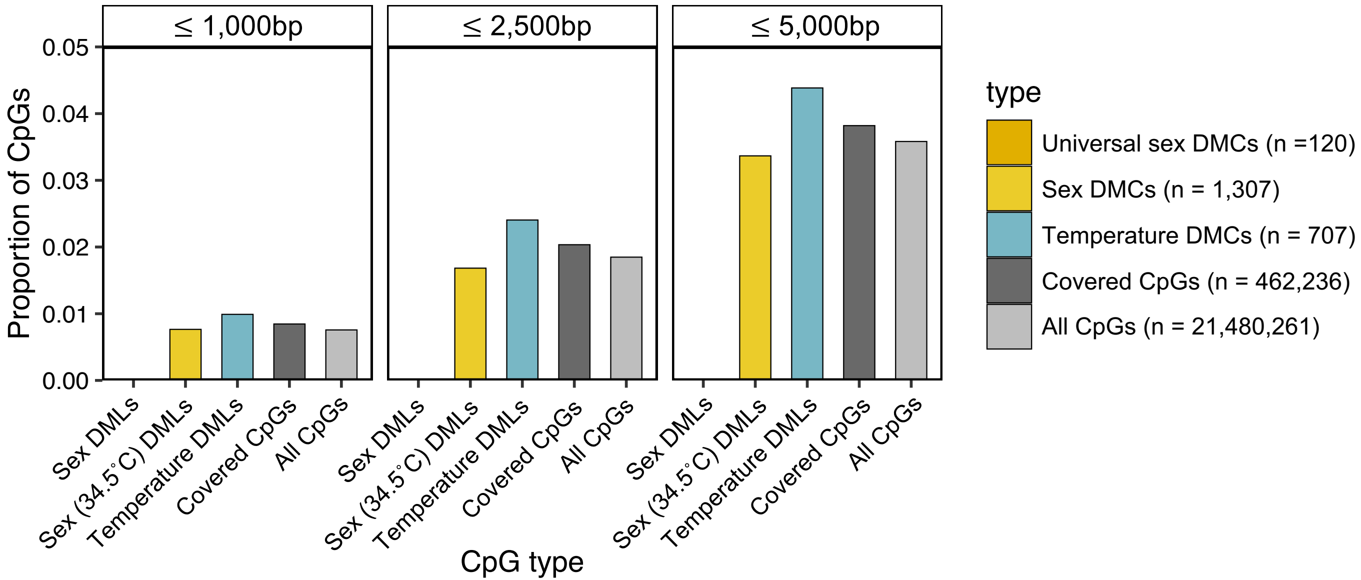

Figure S2. Associations between differentially methylated loci and putative estrogen-response elements. Proportion of CpGs within 1kb, 2.5kb, and 5kb of putative estrogen-response element. Asterisks indicate significant difference in the proportion of covered CpGs in a context category compared to all the CpGs in the alligator genome at an FDR threshold of 10%.

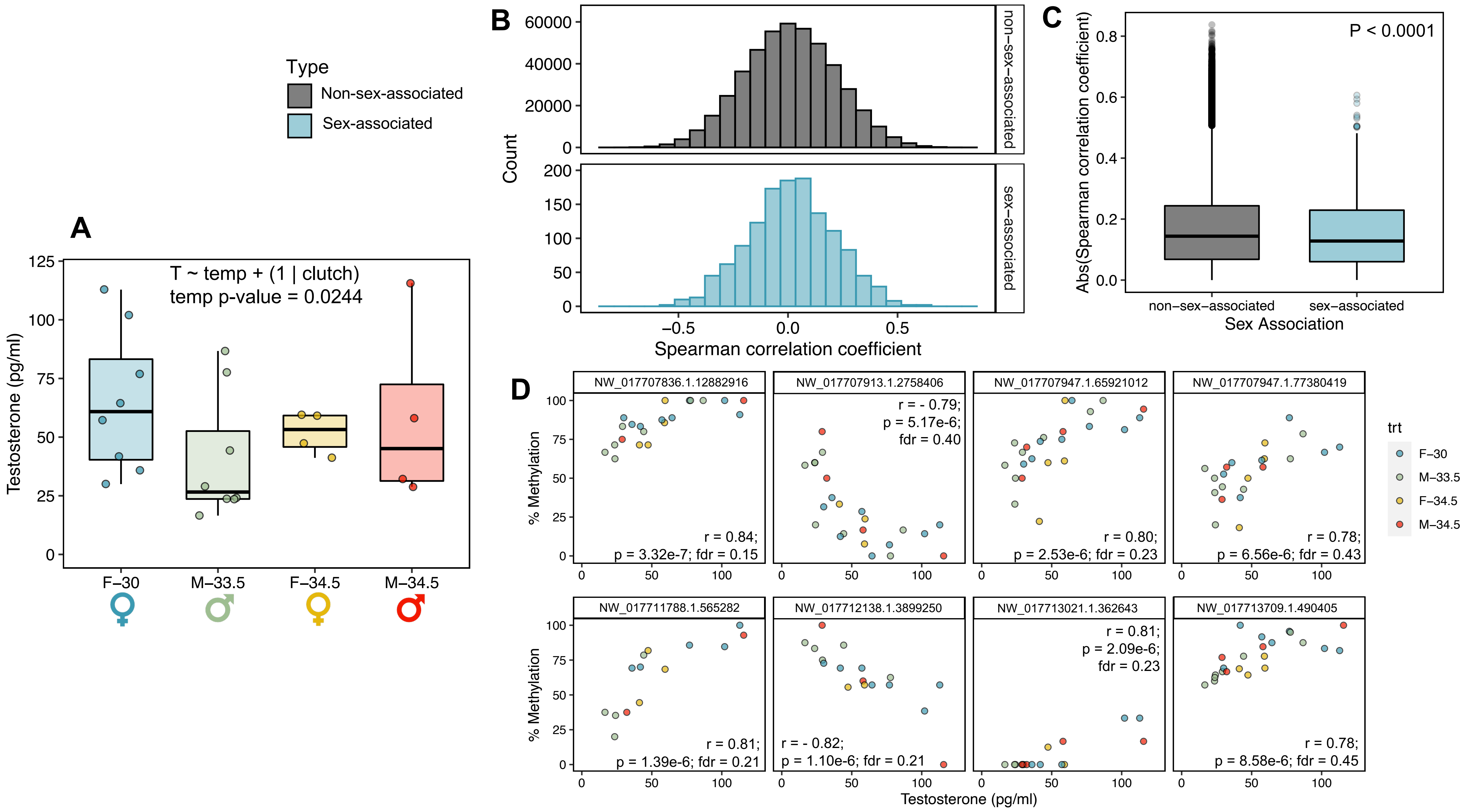

Figure S3. Correlations between plasma testosterone (T) concentrations and genome-wide DNA methylation patterns. (A) Boxplot comparing plasma T concentrations between treatment groups. (B) Histograms of Spearman correlation coefficients for T correlations, with panels separating loci not identified as sex-associated via pairwise comparisons (top) from loci identified as sex-associated differentially methylated cytosines (DMCs; bottom). (C) Boxplot comparing absolute Spearman correlation coefficients between sex-associated DMCs and all other loci. Sex-associated loci collectively exhibited a significantly lower absolute Spearman correlation with plasma T compared to loci not identified as sex-associated. (C) Scatterplots depicting the relationship between percent methylation and plasma T concentration for the loci exhibiting the top 8 most significant correlations. For boxplots, central line depicts median, box depicts interquartile range (IQR), vertical lines depict the maximum and minimum values, and values greater or less than 1.5*IQR are depicted as points.

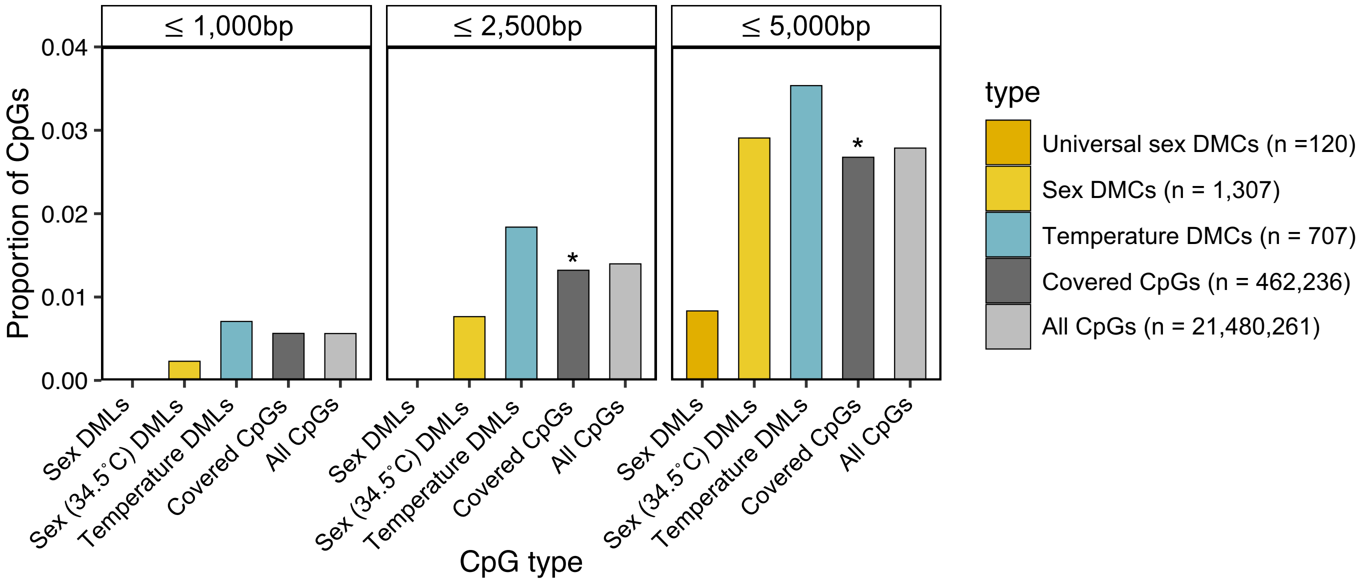

Figure S4. Associations between differentially methylated loci and putative androgen-response elements. Proportion of CpGs within 1kb, 2.5kb, and 5kb of putative androgen-response element. Asterisks indicate significant difference in the proportion of covered CpGs in a context category compared to all the CpGs in the alligator genome at an FDR threshold of 10%.
